# Therapeutic Potential of 1,8-Dihydroanthraquinone Derivatives for Breast Cancer

**DOI:** 10.3390/ijms242115789

**Published:** 2023-10-31

**Authors:** Estera Okon, Katarzyna Gaweł-Bęben, Agata Jarzab, Wojciech Koch, Wirginia Kukula-Koch, Anna Wawruszak

**Affiliations:** 1 Department of Biochemistry and Molecular Biology, Medical University of Lublin, 20-093 Lublin, Poland; estera.okon@umlub.pl (E.O.); agata.jarzab@umlub.pl (A.J.); 2 Department of Cosmetology, University of Information Technology and Management in Rzeszów, 2 Sucharskiego, 35-225 Rzeszów, Poland; kagawel@wsiz.edu.pl; 3 Department of Food and Nutrition, Medical University of Lublin, 4a Chodzki Str., 20-093 Lublin, Poland; wojciech.koch@umlub.pl; 4 Department of Pharmacognosy with Medical Plants Garden, Medical University of Lublin, 1 Chodzki Str., 20-093 Lublin, Poland

**Keywords:** 1,8-dihydroanthraquinone derivatives, breast cancer, natural products, biological activity, emodin, aloe-emodin, hypericin, chrysophanol, rhein, physcion

## Abstract

Breast cancer (BC) is the most common malignancy among women worldwide. In recent years, significant progress has been made in BC therapy. However, serious side effects resulting from the use of standard chemotherapeutic drugs, as well as the phenomenon of multidrug resistance (MDR), limit the effectiveness of approved therapies. Advanced research in the BC area is necessary to create more effective and safer forms of therapy to improve the outlook for individuals diagnosed with this aggressive neoplasm. For decades, plants and natural products with anticancer properties have been successfully utilized in treating various medical conditions. Anthraquinone derivatives are tricyclic secondary metabolites of natural origin that have been identified in plants, lichens, and fungi. They represent a few botanical families, e.g., Rhamnaceae, Rubiaceae, Fabaceae, Polygonaceae, and others. The review comprehensively covers and analyzes the most recent advances in the anticancer activity of 1,8-dihydroanthraquinone derivatives (emodin, aloe-emodin, hypericin, chrysophanol, rhein, and physcion) applied both individually, or in combination with other chemotherapeutic agents, in in vitro and in vivo BC models. The application of nanoparticles for in vitro and in vivo evidence in the context of 1,8-dihydroanthraquinone derivatives was also described.

## 1. Introduction

Cancer is one of the most common causes of death in the world [1]. This diverse disease is a multistage process that is initiated by genetic changes occurring in normal cells, leading to their transformation into cancer cells. Transformed cancer cells are characterized by uncontrolled growth, the ability to invade nearby tissues, and to create distant metastases [2]. Despite significant progress in cancer treatment, including the emergence of new molecule-based therapies, treatment options for cancer patients remain limited due to serious adverse effects and the phenomenon of multidrug resistance (MDR) [3]. Bioactive substances of natural origin may interfere with the process of carcinogenesis by modifying the behavior of neoplastic cells and affecting signaling pathways that have been improperly activated or inhibited [2].

Anthracene and its derivatives have been the subject of extensive research over the years due to their interesting photochemical and bioactivities [4]. They are present in Liliaceae, Fabaceae, Rubiaceae, Polygonaceae, Rhamnaceae, and Scrophulariaceae families. Within the various chemical classes of natural products, anthraquinones are characterized by their extensive structural diversity, significant biological activity, and relatively low toxicity. It has been demonstrated that anthracene derivatives display anti-inflammatory, immunomodulatory, or antineoplastic activities. Anthraquinones inhibited growth of breast, lung, colon, prostate, cervix, or leukemia cancer cells [5].

In the manuscript, the most recent advances in the anticancer activity of 1,8-dihydroanthraquinones, e.g., emodin, aloe-emodin, hypericin, chrysophanol, rhein, and physcion, applied both individually, or combined with other chemotherapeutic agents, in in vitro and in vivo breast cancer (BC) models were comprehensively described.

## 2. Molecular Basis of Breast Cancer

Breast cancer (BC) is the most common malignancy among women worldwide with approximately 2.3 million new cases detected in 2020 (GLOBOCAN 2020). Unfortunately, the rates of BC incidence and mortality are still rising [6,7].

**Figure 1:**
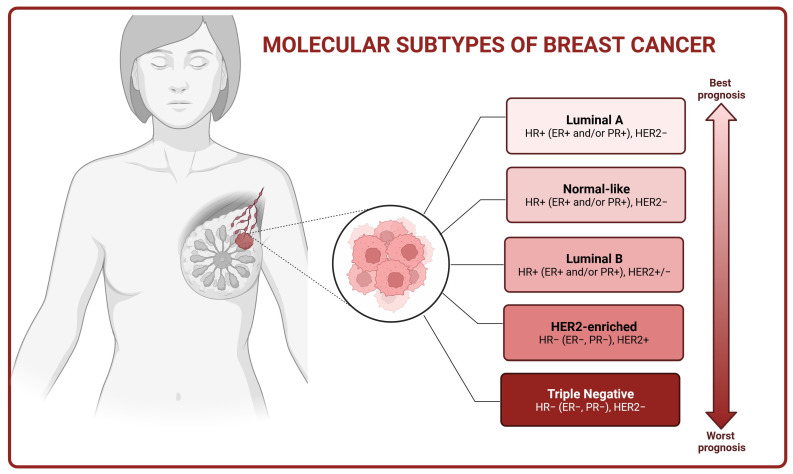
Molecular subtypes of breast cancer (HR—hormone receptor, ER—estrogen receptor, PR—progesterone receptor, HER2—human epidermal growth factor receptor 2). The figure was created with BioRender.com (Toronto, Ontario, Canada) (accessed on 28 September 2023).

Classical immunohistochemistry markers, such as estrogen (ER), progesterone (PR), and human epidermal growth factor (HER2) receptor expression, along with clinicopathological factors like tumor size, grade, and nodal involvement, have traditionally been used for therapy selection and predicting disease progression. However, the widespread use of high-throughput gene expression analysis techniques has revealed that the response of cancer cells to treatment is not solely determined by anatomical prognostic factors but rather by the internal molecular characteristics of BC. Therefore, five distinct molecular subtypes of BC have been identified, including luminal A, luminal B, HER2-overexpressed, triple-negative breast cancer (TNBC), and normal-like (Figure 1) [8].

BCs classified as luminal A exhibit expression of ERs and/or PRs, lack of HER2 expression, and low value of Ki-67 cell proliferation index. This subtype of BC depicts a positive response to endocrine therapy and generally has the best prognosis [9]. In turn, luminal B BC (ER+ and/or PR+, HER2+/−) is associated with a less favorable prognosis than the luminal A subtype and presents a higher proliferating index value. Hormonal therapy along with chemotherapy can be beneficial for the treatment of patients suffering from these tumors [10,11]. The HER2-positive subtype accounts for approximately 10–15% of BCs and is characterized by HER2 overexpression alongside the absence of ER and PR. These tumors tend to grow more rapidly than the luminal subtypes, but the prognosis has significantly improved with the introduction of HER2-targeted therapies [11]. TNBC is defined as a type of BC with a lack of ER, PR, and HER2 protein expression. TNBC represents the most aggressive subtype of BC and is associated with a poor prognosis. Currently, treatment options for TNBC are primarily restricted to surgical intervention, adjuvant chemotherapy, and radiotherapy [12].

One of the most important challenges associated with conventional treatments are the serious side effects and MDR phenomenon. Advanced research in the BC area is necessary to create more effective and safer forms of therapy to improve the outlook for individuals diagnosed with this aggressive neoplasm [12]. Numerous research efforts are now exclusively dedicated to discovering alternative forms of treatment (including natural products) for BC [13]. Promising compounds with proven anticancer properties are 1,8-dihydroanthraquinone derivatives.

## 3. Characteristics of Anthracene Derivatives

Anthracene derivatives are tricyclic secondary metabolites of natural origin which have been identified in plants, lichens, and fungi [14]. They were known since the Antiquity as natural pigments. More than 700 structures of these colored molecules have been described so far, with around one-third in different organs of plants: flowers, fruits, roots, or rhizomes [15] representing a few botanical families, like Rhamnaceae, Rubiaceae, Fabaceae, Polygonaceae [5], and others (Table 1).

**Figure 2:**
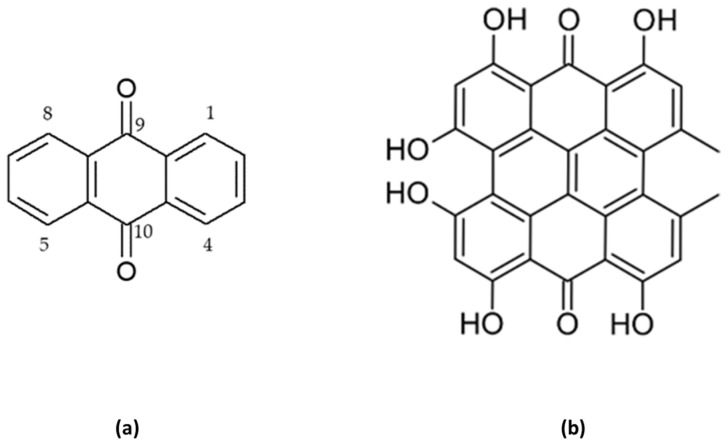
The chemical structures of an anthraquinone (**a**) and hypericin (**b**).

Anthracene derivatives may occur in a reduced or oxidized form. The former are less diverse and are known to be present in fresh plant material as anthrons or anthranols. In turn, oxidized forms of anthracene derivatives, namely, anthraquinones, form a very diverse group of metabolites that are also milder in action. They can be present in fresh plant material, but also, they are formed during drying at 105 °C degrees or storage of bioactive plant material for 1–2 years from the reduced forms. Anthraquinones—the largest group of anthracene derivatives—in their chemical structure contain an anthracene moiety that is substituted with two ketone groups at the positions 9 and 10 forming a 9,10-dioxoanthracene or 9,10-anthracenedione (Figure 2). Additionally, some structures may contain two hydroxyl groups at C-1 and C-8 carbon atoms whose presence induces laxative effects of the compounds. Due to the solubility issues, these metabolites are often present in glycosylated forms in plants to be better dissolved in aqueous solutions of plant cells [16].

Two metabolic pathways are proposed for the biosynthesis of anthraquinones. One of them includes the cyclization of octa-β-ketoacyl-CoA that is produced by the addition of acetyl-coenzyme A to three groups of malonyl-coenzyme A [17]. The other is related to the shikimic acid pathway where an addition of succinoylbenzoic acid to mevalonic acid occurs [18].

**Figure 3:**
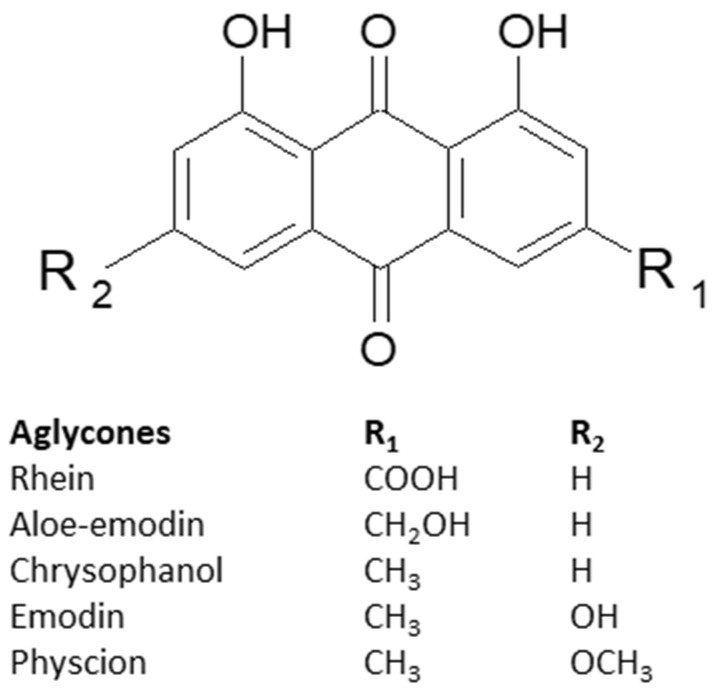
The chemical structures of the main subgroups of anthraquinones.

In the scientific literature, the 1,8-dihydroxyanthraquinones (1,8-OH-AQ) that were proven to exhibit laxative effects and used in traditional medicine are the most recognizable compounds that belong to anthracene derivatives. Differently substituted aglycons of 1,8-OH-AQ can be divided into a few subgroups. So far, the derivatives of emodin, aloe-emodin, physcion, chrysophanol, and rhein have been described in the most thorough manner (Figure 3) [17,18].

An interesting example of a polymeric 1,8-OH-AQ is hypericin. This compound contains two anthracene moieties that are stacked at each other, forming a structure of naftodianthrone by means of oxidative coupling, with additional substituents like hydroxyl or ketone groups. Hypericin and their derivatives, like pseudohypericin or isohypericin, are strong photosensitizers and play a pivotal role in anticancer photodynamic therapy (Figure 2) [19].

Because of these characteristics, anthraquinones as interesting molecules have attracted the attention of chemists who elaborated the total synthesis of many derivatives of naturally occurring structures. Many of them can now be used as chemotherapeutics in the treatment of cancer. Among the approved drugs with elaborated synthetic production, we can list doxorubicin, daunorubicin, idarubicin, epirubicin, valrubicin, mitoxantrone, pixantrone, and others [19].

Considering the structural differences between the anthracene derivatives and the number of publications on their anticancer activity, the aim of this review is to put together the information on 1,8-dihydroxyanthraquinones as potential drug candidates for the treatment of BC, their mechanisms of action, and potential synergistic effects with other commercially available chemotherapeutics. This subgroup of anthracene derivatives is well studied and certainly deserves attention in terms of its anticancer properties.

Further below, a detailed biological potential of emodin, aloe-emodin, hypericin, chrysophanol, rhein, and physcion is presented.

## 4. Anticancer Activity of 1,8-Dihydroanthraquinone Derivatives in Breast Cancer In Vitro Models

### 4.1. Emodin

Of all anthraquinones, the use of emodin in BC therapy has been most extensively described in the scientific literature. In vitro studies confirmed anticancer properties of emodin towards several types of human BC cell lines, including BCap-37 [20], MCF-7 [21], MDA-MB-453 [22], MDA-MB-231 [23], and GILM2 human BC cells obtained from lung metastasis [24]. The use of different types of cell models helped uncover the molecular mechanisms behind the therapeutic potential of emodin in the treatment of BC.

Emodin has been characterized as a strong proapoptotic agent. It has been showing that the treatment of the human BCaP-37 BC cell line with emodin at 20 and 50 µM for 48 h induced morphological characteristics for apoptosis, decreased in Bcl-2/Bax ratio, and increased cytosolic cytochrome c concentration. These changes indicate the involvement of the mitochondrial signaling pathway in the emodin-induced apoptosis [20]. Subsequent studies by the same researchers revealed that emodin regulates the expression of about 30 specific genes in BCap-37 cells, including insulin-like growth factor 2 (IGF-2, downregulated by emodin) and protein p21 (upregulated by emodin) [25]. In BCap-37 and ZR-75-30 BC cell lines, emodin reduced the expression of Bcl-2 (B-cell CLL/lymphoma 2) and increased levels of cleaved caspase-3, PARP (poly (ADP-ribose) polymerase), p53, and Bax, resulting in dose- and time-dependent proapoptotic effects [26].

In MDA-MB-231 cells, emodin was significantly cytotoxic at concentrations of 10–80 µM following 24, 48, and 72 h treatment. Emodin has also been shown to dose-dependently inhibit the migration and invasion of MDA-MB-231 cells and thus may be considered effective in the prevention of BC metastasis. Enzyme-linked immunosorbent assay (ELISA) and Western blot analyses revealed that the anti-metastatic potential of emodin results from the downregulation of proteolytic enzymes involved in the degradation of extracellular matrix components, triggering metastasis. Emodin at 40 and 80 µM decreased the production of matrix metalloprotease (MMP) 2 and 9, uPA, and uPAR in MDA-MB-231 cells and reduced the levels of p38 and ERK kinases [23].

Emodin inhibited the growth of MCF-7 BC cells with the half-maximal inhibitory concentration (IC_50_) = 7.22 µg/mL and demonstrated a dose-dependent inhibitory effect on the colony formation of MCF-7 cells with IC_50_ = 7.60 µg/mL. Single-strand DNA breakage and DNA fragmentation, considered as hallmarks of apoptosis, were observed in MCF-7 cells treated with emodin. The proapoptotic effect of emodin is more likely mediated through modulation of the expression of apoptosis-related genes, such as Fas ligand (FasL), myeloid cell leukemia sequence 1 (MCL1), glyceraldehyde-3-phosphate dehydrogenase (GAPDH), Bcl2-associated X protein (Bax), cyclin D1 (CCND1), and v-myc myelocytomatosis viral oncogene homolog (C-MYC). The 72 h emodin treatment significantly upregulated the expression of FASL and downregulated the expression of MCL1, CCND1 and C-MYC genes as compared with untreated control cells [27]. Anticancer properties of emodin are strongly dependent on its tyrosine kinase inhibitory potential. Zhang et al. described that emodin could act as a tyrosine kinase inhibitor, decrease the activity of HER-2/neu tyrosine kinase in MDA-MB-453 cells, inhibit the growth of cancer cells, induce the production of lipid droplets, and promote the mature differentiation of BC cells. One of the emodin derivatives, 10-(4-acetamidobenzylidene)-9-anthrone (DK-V-47), was more effective than emodin in repressing the tyrosine phosphorylation of p185neu and in inhibiting the proliferation and transformation of HER-2/neu-overexpressing human BC cells. DK-V-47 was also more potent than emodin in suppressing transformation phenotypes of activated HER-2/neu-transformed 3T3 fibroblasts, including anchorage-dependent and -independent growth and metastasis-associated properties. These results clearly indicated that the inhibition of p185neu tyrosine kinase by both emodin and DK-V-47 suppressed the HER-2/neu-associated phenotype of BC cells, including their ability to metastasize [28]. The same group has reported that the combination of emodin (20 µM) and paclitaxel (1 µM) synergistically inhibited the anchorage-dependent and -independent growth of HER-2/neu-overexpressing MDA-MB-361, BT-474, MDA-MB-231, and MDA-MB-435 BC cells in vitro. The mechanism is related to the reduction of tyrosine phosphorylation of HER-2/neu, suggesting that HER-2/neu inhibition is one of the important approaches of emodin in BC treatment [29].

Emodin also sensitizes HER2/neu-overexpressing cancer cells to chemotherapeutic agents, including cisplatin, doxorubicin, etoposide, and paclitaxel [30]. Emodin also effectively inhibited the growth of MDA-MB-435 cells with low HER-2/neu expression by decreasing tyrosine kinase [31]. Recently, it has also been confirmed that emodin significantly reduced the phosphorylation levels of ERK1/2 and AKT but not p38 MAPK in MDA-MB-231 cancer cells. Emodin inhibited BC cell proliferation and invasion through the serine/threonine kinase (AKT) signaling and extracellular-regulated protein kinase (ERK) pathways [32].

Using virtual screening, Zhang et al. found that emodin is an effective aromatic hydrocarbon receptor (AhR) agonist. Subsequent in vitro experiments also found that the expression levels of AhR and cytochrome P450 1A1 (CYP1A1) in MCF-7 cells were significantly upregulated by emodin treatment, suggesting that the antitumor effects of emodin against BC might be related also to the AhR-CYP1A1 signaling pathway [33].

Emodin has been implicated in the regulation of estrogen signaling in BC cells. Sui et al. found that emodin inhibited estrogen-induced proliferation of MCF-7 and MDA-MB-231 cells, promoted apoptosis, and arrested the cell cycle in the G0/G1 phase by downregulating the expression of cyclin D1 Bcl-2 and estrogen receptor (ER) α proteins [34]. In addition, emodin induced breast cell apoptosis and proliferation through ERα inhibition [26,35].

The effects of emodin on MDA-MB-231 and MDA-MB-453 human TNBC cell lines alone or co-cultured with human adipocytes were investigated. The results showed that emodin inhibited TNBC proliferation and invasion more efficiently when co-cultured with adipocytes by downregulating the level of CC-chemokine ligand 5 (CCL5) in adipocyte supernatants; inhibiting the expression level of protein kinase B (AKT); and activating glycogen synthase kinase-3i (GSK3) and β-catenin. This led to the suppressed expression of EMT- and invasion-associated markers, including vimentin, snail, matrix metalloproteinase (MMP)-2, and MMP-9, and upregulation of E-cadherin, contributing to the inhibition of invasion [36].

The anticancer properties of an emodin azide methyl anthraquinone derivative (AMAD), extracted from the nature giant knotweed rhizome of traditional Chinese herbs, were investigated. The IC_50_ of AMAD was 9.06 ± 0.95 μmol/L for MDA-MB-453 cells, whereas for normal mouse fibroblast NIH3T3 cells the IC_50_ was >100 μmol/L. Apoptotic induction was associated with a collapse of the mitochondrial membrane potential and activated caspase cascade involving caspase-8, caspase-9, caspase-3, and PARP cleavage in a concentration-dependent manner. AMAD also effectively increased the cleavage of Bid, a BH3 domain-containing proapoptotic Bcl-2 family member, and induced the subsequent release of cytochrome c from mitochondria into the cytosol [22]. In another study, Yan et al. showed that in cancer cells overexpressing HER2/neu, overtreatment with AMAD inhibited MAPK and PI3K/AKT-dependent signaling pathways, leading to growth inhibition and induction of apoptosis. It was shown for the first time that emodin treatment impairs the binding of HER2/neu to Hsp90, intracellular redistribution, enhanced ubiquitinylation, and subsequent proteasomal degradation of HER2/neu, which may represent a novel approach for the targeted therapy of HER2/neu-overexpressing cancers [37].

The proapoptotic potential of emodin was demonstrated in vitro at concentrations of >10 µM. At lower concentrations (<10 µM), emodin had no or very mild effect on the viability of invasive BC MDA-MB-468 and MDA-MB-435 cell lines in vitro, but it was significantly influencing their invasive potential by specifically antagonizing the adenosine 5’-triphosphate (ATP)-gated Ca^(2+)^-permeable channel P2 × 7 receptor (P2X7R) [38]. P2X7R is highly expressed in many tumors and cancer cells and has been found to play an important role in the migration and invasion of metastatic tumor cells [39]. Studies by Jelassi et al. showed that an increase in gelatinolytic activity, in cancer cell invasiveness in vitro and cell morphology changes induced by ATP, are prevented by 1 µM emodin [38].

Emodin has also been shown to prevent the tumor-promoting interactions between BC cells and tumor-associated macrophages (TAMs). TAMs are the most abundant leucocytes in the tumor microenvironment (TME), responsible for remodeling of TME in response to various signals including those from cancer cells [21]. Emodin reduced the infiltration of macrophages to the tumor and their subsequent M2-like polarization in tumor-bearing mice injected intraperitoneally with 40 mg emodin/kg/day and thus ameliorated the immunosuppressive state of TME. Moreover, emodin treatment also suppressed the response of macrophages to the tumor-derived soluble factors. The treatment of mouse peritoneal macrophages with conditioned medium from EO771 human adenocarcinoma increased the expression of M2 macrophage receptor CSFr1, histone demethylase JMJD3, and adhesion molecule ICAM-1, whereas emodin reversed this effect [40,41]. Treatment of macrophages obtained from mice with emodin suppressed their migration towards tumor-conditioned medium. Emodin decreased phosphorylation of STAT6 and expression of C/EBPβ, two crucial signaling events in macrophage M2 polarization [42]. Emodin suppressed TGF-β1 production in BC cells and macrophages and attenuated TGF-β1 or macrophage-induced epithelial–mesenchymal transition (EMT) and cancer stem cell (CSC) formation of BC cells. Emodin was also shown to reverse the suppressive effect of tumor microenvironment on the activation of T cells [43].

The influence of emodin on the ETM transition induced by fibroblasts isolated from the tissues of TNBC patients was investigated. Using an in vitro co-culture model, interface zone fibroblasts (INFs) or cancer-associated fibroblasts (CAFs) induced EMT and promoted cancer cell migration in epithelial BT20 cells. Interestingly, we found that emodin inhibited EMT programming and phenotype in epithelial BT20 cells induced with INFs- and CAFs-conditioned medium [44].

Emodin is also a promising antiangiogenic factor. Kwak et al. demonstrated that emodin inhibited VEGF-A-induced proliferation, migration, invasion, and tube formation of Human Umbilical Vein Endothelial Cells (HUVEC) in vitro. Moreover, emodin also impaired basic fibroblast-growth-factor-induced proliferation and migration of HUVECs and VEGF-A-induced tube formation of human dermal microvascular endothelial cells. Emodin arrested growth of VEGF-A-stimulated HUVECs at the G0/G1 phase of the cell cycle through downregulation of cyclin D1 and E. Emodin blocked VEGF-A-induced tyrosine phosphorylation of VEGF receptor KDR/Flk-1 and downstream signaling molecules including FAK, ERK1/2, p38 MAPK, AKT, and endothelial nitric oxide synthase, showing its potential antiangiogenic activity [45]. Emodin also attenuated cancer cell metastasis and angiogenesis in vitro via MMPs and vascular endothelial growth factor receptor 2 (VEGFR2) inhibition, which may be associated with the downregulation of the *Runx2* [46], a transcription factor which is one of the members in the *Runx* gene family encoding proteins homologous to Drosophila Runt and a potential target for inhibition of metastatic growth of BC cells [47]. Studies have demonstrated that atypical expression and function of Runx2 are associated with the formation of bone metastasis in BC [46].

Zou et al. found that emodin increased the expression of SerRS, which is a strong transcriptional inhibitor of VEGFA in TNBC cells. In addition, a direct target of emodin—nuclear receptor corepressor 2 (NCOR2)—has been identified. When NCOR2 binds to emodin, it is released from the SerRS promoter, resulting in the activation of SerRS and inhibition of VEGFA transcription [48].

Emodin might also be effective in the prevention of multidrug resistance of BC cells and the treatment of drug-resistant BC. Fu et al. reported that emodin at 10 μg/mL downregulated the expression of DNA excision repair protein ERCC-1 and inhibited doxorubicin (DOX)–cisplatin resistance in MCF-7 cells [49]. Zu and co-workers showed that emodin (20 μM) increased the sensitivity of MCF-7 BC cells to chemotherapy and promoted 5′fluorouracil-induced apoptosis and cellular senescence. The mechanism was related to the inhibition of NRARP, and silencing NRARP blocked the effect of emodin on MCF-7 cells [50]. Li et al. reported that DOX combined with emodin can improve the sensitivity of MDA-MB-231 and MCF-7 cells to chemotherapy, and the mechanism is closely related to increasing γH2A in cancer cells and regulating AKT1-mediated DNA damage [50]. These data are particularly promising as DOX is a commonly used chemical drug against BC, but the rapid emergence of drug resistance is a major culprit limiting its clinical use [51].

The other approach important for emodin application in BC therapies is the delivery of the drug to BC cells. Wang et al. used the high-pressure homogenization method to produce emodin-loaded solid lipid nanoparticles (E-SLNs), characterized by stable particle size of 28.6 ± 3.1 nm, good drug entrapment efficiency, and relatively good over long-time (4 months) storage. In vitro cytotoxicity of E-SLNs (5–30 µM) towards human BC cell lines MCF-7 and MB-MDA-231 was significantly higher than free emodin, whereas unloaded SLNs were not cytotoxic for both cell lines [52]. Liu et al. investigated the therapeutic potential of polymer lipid hybrid nanoparticles loaded with emodin (E-PLNs), obtained by the nanoprecipitation method. The average particle size of the E-PLNs was 122.7 ± 1.79 nm, and the encapsulation rate was 72.8%. Compared with free emodin, E-PLNs showed greater toxicity towards MCF-7 cells by promoting emodin uptake and inducing apoptosis [53]. Emodin and emodin-containing scaffolds were also shown as cytotoxic toward the GILM2 human BC cell line obtained from lung metastasis [24].

Several in vitro studies on BC models are not limited to emodin alone but also include its combination with other natural compounds: berberine, thymoquinone, daunorubicin, and curcumin. These combined treatments seem to achieve better antitumor effects, which may become an effective strategy for BC therapies [54,55,56,57].

### 4.2. Aloe-Emodin

The effect of aloe-emodin (AE) on human BC cell proliferation and survival has also been investigated using various in vitro models. AE in the concentration range of 10 to 50 μM showed estrogen-like activity, by increasing the proliferation of human ER-positive MCF-7 cells and was investigated. The observed effects were attenuated by co-treatment with an ER antagonist—fulvestrant. Using another experimental condition, AE showed significant cytotoxicity towards human ER-positive MCF-7 and ER-negative MDA-MB-231 BC cells by inducing mitochondria-independent apoptosis by activating the caspase-8 pathway [58]. The cytotoxic effect of AE was also observed using MDA-MB-231 cells [59].

Aloe-emodin was proposed as one of the components of aloe vera extract responsible for its cytotoxic activity for the MCF-7 human BC cell line due to its particularly strong calculated binding affinity toward ERα (−8.8 kcal/mol) as compared to the standard drug tamoxifen (−6.4 kcal/mol) [60].

Effective application of aloe-emodin as an anticancer drug is limited due to its poor water solubility and low bioavailability. These difficulties may be overcome by loading aloe-emodin onto various carriers. Freag et al. designed and produced surface-functionalized polyethylene glycol liquid crystalline nanoparticles (PEG-LCNPs) of aloe-emodin (AE) to enhance its water solubility and increase clinical relevance. AE-PEG-LCNPs were characterized by particle size of 190 nm and zeta potential of −49.9 and increased serum stability. Moreover, nanoparticles were also stable following sterilization by autoclaving and γ-radiation. In vitro studies using human breast adenocarcinoma cell line (MCF-7) showed that, following 48 h of incubation, the half-maximal inhibitory concentration of AE-PEG-LCNPs was 3.6-fold lower than free AE, and their cellular uptake was 3-fold higher than free AE following 24 h treatment [61]. Chen and co-workers synthesized AE-loaded solid lipid nanoparticles (AE-SLNs) characterized by stable particle size at 88.9 ± 5.2 nm, promising drug entrapment efficiency (EE) of 97.71 ± 0.5% and good stability regarding zeta potential as high as −42.8 mV. In vitro cytotoxicity studies showed that AE-SLNs are more cytotoxic for MCF-7 BC cells than free AE solution and have no significant toxicity towards noncancerous human mammary epithelial MCF-10A cells. Incubation of MCF-7 cancer cells with AE-SLNs also increased the number of apoptotic cells and AE uptake when compared with free AE treatment [62].

### 4.3. Aloin A & B

**Figure 4:**
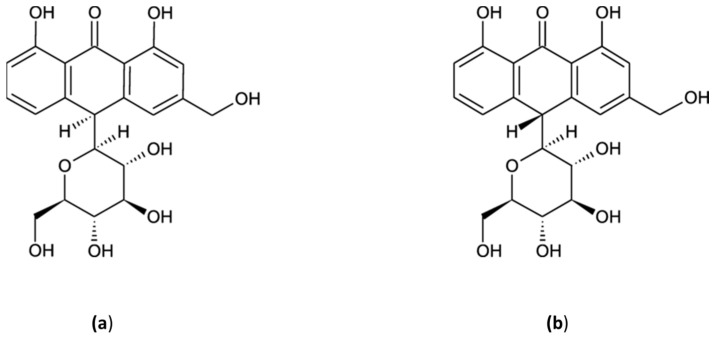
The chemical structures of aloin A (**a**) and aloin B (**b**).

Aloin (Figure 4) might be involved in the suppression of BC cell growth in vitro by DNA intercalating activity, as predicted using computational screening [63]. Esmat et al. investigated the influence of aloin treatment (20 and 60 µg/mL) on the nuclear DNA ploidy of ER- and prostaglandin receptor (PgR)-positive breast tumor cell line T47D [64]. Change in DNA content (ploidy) has been described in a variety of tumors and it might be related to the patient survival rate and response to the treatment [65]. Aloin treatment has not changed the DNA ploidy but caused a significantly dose-dependent increase of the BC cells in the S phase and the appearance of cells cycling at a higher ploidy level (>G2M). These results suggest that aloin does not inhibit the initiation of DNA synthesis and that cells replicated a full complement of DNA but had difficulty in the M phase. The mechanism of aloin cytotoxic action might involve inhibition of topoisomerase II, an enzyme known for its ability to alter DNA supercoiling, essential for the survival of eukaryotic cells [66].

The cytotoxic potential of aloin using two human BC cell lines, MCF-7 and SKBR-3, characterized by the lack or presence of erbB-2-topoIIα gene co-amplification, respectively, was described. The MCF-7 cell line was more sensitive to aloin treatment than the SKBR-3 cell line, as demonstrated by MTT and clonogenic assays. Aloin at higher concentrations induced apoptosis, inhibited topo IIα protein expression, and downregulated cyclin B1 protein expression in the MCF-7 cell line, whereas erbB-2 protein expression was not affected. Topo IIα protein expression was only mildly decreased in SKBR-3 cell line, only at higher concentrations. It suggests that the aloin cytotoxic effect is regulated by more than one mechanism, depending on the dose level and phenotype of breast tumor cells [67].

Aloin was also shown to significantly decrease the mammosphere formation at 50, 100, and 200 µM, indicating the cytotoxic effect of these compounds on the subpopulation of cancer stem cells (CSC) [68].

### 4.4. Physcion

Anti-breast cancer properties of physcion were demonstrated in MCF-7 cells due to an influence on the mitochondrial apoptotic pathway mostly by impact on oxidative stress [69]. Physcion was observed to stimulate apoptosis via activation of caspase pathways. Moreover, it caused the accumulation of reactive oxygen species (ROS) which disturbed the proper functioning of mitochondria [70]. Other studies showed that physcion influences the level of pro- and antiapoptotic proteins. The expression of Bax protein was increased, but Bcl-xL and Bcl2 were diminished. The creation of the Bcl-2/Bax heterodimer demonstrates a proapoptotic impact by inhibiting Bcl-2, leading to the production of ROS and the modulation of MMP levels [71]. The excess of arising ROS leads to the stimulation of the caspase cascade, which is involved with apoptosis activation [72]. Intracellular ROS play the role of second messengers for certain growth factors to have an impact on cytokines and hormones, resulting in mitochondrial oxidative harm and programmed cell death through the Nrf2 signaling pathway, subsequently causing additional ROS release from the mitochondria [71].

The current research findings indicate that physcion suppressed the growth of MDA-MB-231 cells in a manner that depended on the dosage used in MDA-MB-231 human BC cells. In cells exposed to physcion, there was a reduction in the number of cells in the S phase, while there was an increase in the number of cells in the G0/G1 phase compared to untreated cells. These findings suggest that physcion prompted cell cycle advancement by causing G0/G1 phase arrest [73]. The advancement of the cell cycle involves a step-by-step activation of CDKs, which require interaction with their respective regulatory cyclins for activation. Specifically, the cyclin D/CDK4 complex plays a crucial role in controlling the G0/G1 phase, while the cyclin E/CDK2 and cyclin A/CDK2 complexes are linked to the G1/S transition and the onset of the S phase, respectively [74].

Consequently, the inhibition of cyclin D, cyclin E, cyclin A, CDK2, and CDK4 by physcion contributed to the arrest of the cell cycle at both the G0/G1 and G1/S phases. The CDK inhibitor p21 can hinder cell cycle progression by suppressing the kinase activities of CDKs/cyclin complexes. Moreover, cyclin–CDK complexes deactivate the tumor suppressor Rb by phosphorylating Rb at serine sites. In the case of MDA-MB-231 cells, the expression of p21 was induced by physcion, while pRb was suppressed. These findings suggest that physcion is involved in causing cell cycle arrest at the G0/G1 phase [73].

### 4.5. Rhein

Recent studies demonstrated that modifying the structure of rhein by introducing a benzyloxy group in the place of the 1,8-phenolic hydroxyl group changes the properties of this 4F compound. This indicates that derivative 4F may be a selective anthracycline candidate with low toxicity. Based on this, the effect of derivative 4F on the cancer cells was observed in recent experiments. In recent research, we observed that derivative 4F effectively slowed down cell growth, exhibiting a time- and dose-dependent response. Remarkably, it managed to spare normal breast cells from excessive harm while targeting BC cells [75]. Moreover, its impact on curtailing the proliferation and expansion of BC cells surpassed that of rhein and the control, significantly extending the time it takes for cancer cells to double. This indicates a successful modification of the rhein side chain, suggesting that derivative 4F could be a promising, low-toxicity candidate within the anthracycline class [76]. Another protein which has garnered attention as a potential therapeutic target for tumors due to its role in regulating various signaling pathways governing physiological processes is Rac1 protein, a small signal GTPase. In this research, molecular docking was tested to assess the binding stability between derivative 4F and Rac1, discovering that derivative 4F exhibited stronger interactions by forming more hydrogen bonds and arene-cation bonds with Rac1, along with increased hydrophobic interactions with amino acid residues compared to Rhein and the control inhibitor. This suggests greater stability of the Rac1-derivative 4F complex. To substantiate these findings, a luciferase reporter gene assay was conducted, revealing that derivative 4F effectively reduced Rac1 promoter activity in BC cells in a dose-dependent manner, accompanied by a dose-dependent downregulation of Rac1 protein expression. These results indicate that derivative 4F has practical potential in regulating Rac1 [75].

There are examples of other rhein derivatives that have promising effects in BC treatment. Researchers observed that rhein lysinate (RHL), an analog of rhein, effectively suppressed the phosphorylation of EGFR, MEK, c-Raf, and ERK, while inducing apoptosis in MCF-7, SK-Br-3, and MDA-MB-231 cells. This investigation also revealed that rhein could enhance the sensitivity of BC cells to taxol by reducing phospho-epidermal growth factor receptor (p-EGFR) levels, shedding light on its potential to mitigate drug resistance issues in BC cells [77]. Other reports indicate that rhein exerts inhibitory effects on Akt phosphorylation, promoting the activation of FOXO3a. This activation, in turn, enhances the proapoptotic protein Bim’s activity, resulting in caspase protein cleavage and the subsequent initiation of apoptosis within MCF-7 cells. Rhein’s capabilities extend to the inhibition of NF-κB activation and its downstream targets, namely, HIF-1α and VEGF165, in BC cells such as MCF-7 and MDA-MB-435. Furthermore, in vitro studies demonstrated that rhein effectively suppresses HER-2 protein phosphorylation in SK-Br-3 cells, suggesting its potential in the development of therapies tailored for HER-2-positive BC [77].

### 4.6. Chrysophanol

In the research conducted on BC cell lines, it was discovered that chrysophanol effectively suppressed the growth of BT-474 and MCF-7 cells. This suppression occurred by inducing the production of ROS and triggering endoplasmic reticulum stress. These effects were mediated through the activation of unfolded protein response (UPR) regulatory proteins such as PERK, eIF2α, GADD153, and IRE1α, involving the Akt and MAPK pathways [78]. Chrysophanol also initiated a nonapoptotic form of cell death via the mitochondrial cell death pathway. Anticancer properties of chrysophanol regarding arresting cancer cells in the S phase of the cell cycle due to reductions in proteins like cyclin D, CDK2, and thymidylate synthase were observed in BC MCF-7 and MDA-MB-231 cells [79]. Specifically, chrysophanol suppressed the proliferation of MCF-7 and MDA-MB-231 cells in a concentration-dependent manner by halting the progression of BC cells at the G1-S cell cycle checkpoint [80]. This was achieved by significantly inhibiting the expression of cyclin family proteins, including cyclin D1 and cyclin E, while increasing P21 levels in both cell lines, as confirmed by Western blot analysis and PCR results [81].

Chryspohanol effectively restrained the proliferation of MCF-7 and MDA-MB-231 cells in a dose-dependent manner. Additionally, it induced cell cycle arrest at the G1-S checkpoint, leading to decreased levels of cyclin D1 and cyclin E proteins. Chr also enhanced the apoptotic effects of paclitaxel (PTX) and reduced the expression of Bcl-2. Notably, Chr was observed to deactivate IκB and p65 phosphorylation, which are crucial components of the NF-κB pathway. To confirm the role of NF-κB in chrysophanol’s anticancer effects, an NF-κB inhibitor, PDTC, was employed. In PDTC-treated cells, the impact of chrysophanol on Bcl-2 was less pronounced compared to normal MCF-7 and MDA-MB-231 cells, suggesting that chrysophanol exerts its effects by inhibiting NF-κB activity [80]. These findings align with previous reports indicating that chrysophanol hinders cancer cell growth through NF-κB/cyclin signaling modulation. Furthermore, chrysophanol promoted apoptosis, coinciding with decreased levels of Bcl-2 protein and the cleavage of caspase 3 and PARP. Overall, these results suggest that chrysophanol targets NF-κB/Bcl-2 to suppress BC cell proliferation and enhance sensitivity to chemotherapy, potentially serving as a valuable chemotherapeutic agent against BC cells [80].

It was demonstrated that chrysophanol exhibits anticancer properties when applied to human BT-474 and MCF-7 BC cells. Chrysophanol effectively inhibited the proliferation of these BC cells and induced apoptosis, while sparing normal breast ductal cells. Its influence on BT-474 and MCF-7 cell fate involved the activation of proapoptotic proteins within the mitochondria, the generation of ROS, and the induction of endoplasmic reticulum stress proteins. Additionally, chrysophanol played a role in regulating signaling proteins related to MAPK and PI3K/AKT pathways. Furthermore, unveiled new findings, including the increased presence of proapoptotic proteins (Bax, Bak, and cytochrome c), elevated cytosolic calcium ions and the loss of mitochondrial membrane potential (MMP) in response to chrysophanol treatment in BT-474 and MCF-7 cells. Moreover, chrysophanol triggered the activation of ER stress regulatory proteins, such as PERK, eIF2α, IRE1α, and GADD153, in a dose-dependent manner [78].

### 4.7. Hypericin

In recent studies, the impact of hypericin (HYP) encapsulated within Pluronic F127 (F127/HYP) in photodynamic therapy (PDT) on the MDA-MB-231BC cell line (representing TNBC) was explored compared to normal human breast ductal cells (MCF-10A). The spectroscopic properties of HYP when formulated in F127 copolymeric micelles indicated successful solubilization and suggested the potential of this biocompatible copolymer as a drug delivery system for HYP [82]. The in vitro findings revealed that F127/HYP micelles exhibited potent and selective phototoxic effects on BC cells, demonstrating time- and dose-dependent behavior, while sparing normal cells (MCF-10A). These results align with previous research, where HYP in P123 micelles PDT showed a similar selective impact on MCF-7 cells but not on MCF-10A normal cells [83]. This investigation underscores the robust photodynamic activity of F127/HYP, devoid of dark toxicity, making it a promising option for PDT in the context of breast cancer, particularly TNBC [82].

Furthermore, F127/HYP micelles were observed to accumulate in both the endoplasmic reticulum and mitochondria, leading to cell death via necrosis. The cellular uptake and subcellular distribution of F127/HYP micelles effectively addressed the hydrophobicity issue associated with HYP. Concerning cell death, exposure to F127/HYP PDT induced necrosis in MDA-MB-231 cells. It is known that photosensitizers (PSs) located in mitochondria or the ER typically induce apoptosis, while PSs targeting the plasma membrane or lysosomes can hinder the apoptotic process, potentially leading to necrosis [84]. Overall, these results suggest that F127/HYP micelles hold promise as a valuable platform for the targeted delivery of HYP, offering an effective approach for the treatment of TNBC through PDT.

Photoactivated HYP effectively reduced the mRNA and protein expression of HER2 in both SKBR-3 and MCF-7 cells [85]. It also increased the generation of ROS in MCF-7 and MDA-MB-231 cells. Furthermore, the inhibition of superoxide dismutase-2 (SOD-2) by methoxyestradiol significantly heightened the sensitivity of MCF-7 cells to HYP [86]. In vitro experiments confirmed that HYP and hypericinates could permeate the membrane of MCF-7 cells and accumulate in organelles proximal to the nucleus. Moreover, photodynamic assessments indicated that HYP could impede the formation of cellular colonies, suggesting its potential in preventing tumor recurrence [87]. It was demonstrated that HYP at concentrations of ≥50 μg/mL induces rapid cell death in cancer cells. Recent investigations have highlighted the significance of ADAMTS1 activity in BC development and progression [88]. In the recent research, HYP at concentrations of ≥5 μg/mL also prompted swift cancer cell death, suggesting that hypericin’s inhibitory effect on cell proliferation may be mediated through its tumor-suppressive and cytotoxic properties, primarily involving ADAMTS1 [89].

It has been established that HYP exhibits an antiproliferative impact at lower concentrations, while at higher doses, it induces apoptosis and can disrupt cell mitosis [90]. In a prior study examining the relationship between BC cells and ADAMTS9, hypericin’s potential antitumor effects may be attributed to its interaction with ADAMTS1, ADAMTS3, and ADAMTS9, considering the apoptotic and extracellular effects of ADAMTS9 and the antiangiogenic properties of ADAMTS1 [89].

BC cells release substances that influence pre-osteoclasts, osteoblasts, and bone stromal cells. This stimulation leads to the development of mature osteoclasts that break down bone tissue. Consequently, growth factors are released, further promoting the proliferation of BC cells and perpetuating a harmful cycle of bone degradation [91]. RANKL prompted the formation of many multinucleated osteoclasts initially. However, HYP treatment significantly hindered osteoclast differentiation, leading to a dose-dependent reduction in osteoclast numbers. Notably, the appearance of TRAP-positive osteoclasts occurred after three days of RANKL stimulation, with more mature osteoclasts forming and merging over the subsequent two days. In contrast, HYP treatment consistently inhibited osteoclast differentiation throughout this process [92]. Importantly, previous research demonstrated that HP, at the same doses that inhibited osteoclast differentiation, had no cytotoxic effects [93], which suggests that HP can effectively suppress osteoclast formation in a dose-dependent manner.

To pinpoint when HYP interferes with osteoclastogenesis, HYP (1.2 μM) was introduced to the culture medium on days 0, 1, 2, 3, or 4 of osteoclast differentiation. The most significant inhibitory effects were observed when HYP was administered alongside RANKL treatment, particularly at the outset. In contrast, exposing precursor cells to HYP at later stages (after three days) resulted in less effective suppression. These findings indicate that HYP primarily inhibits early osteoclast differentiation [92]. BC cells can directly influence osteoclast precursor cells to promote osteoclast differentiation. HYP significantly reduced these stimulatory effects induced by BC MDA-MB-231 cells, indicating its inhibitory role in osteoclastogenesis and osteoclast activity driven by these cancer cells [92].

One of the latest studies demonstrated that utilizing gold nanoparticles (AuNPs) as carriers for hydrophobic PSs like HYP enhances the efficacy of PDT, primarily causing apoptosis-mediated cell death. PDT remains an appealing cancer therapy due to its minimally invasive nature and tumor cell selectivity. However, achieving efficient drug delivery to tumor cells is crucial and requires comprehensive exploration. In this research, HYP was attached to AuNPs as carriers to enhance its uptake by MCF-7 BC cells and consequently improve PDT effectiveness. In this investigation, HYP was physically attached to AuNPs via sonication, forming a compound held together by non-covalent bonds. This approach increased the drug’s accumulation within MCF-7 BC cells, consequently elevating PDT efficacy across various concentrations. Therefore, the non-covalent conjugation of HYP with AuNPs holds promise as a strategy for enhancing PDT’s effectiveness in delivering hydrophobic PS drugs [93].

## 5. Anticancer Activity of 1,8-Dihydroanthraquinone Derivatives in Breast Cancer In Vivo Models

The evidence on the anticancer potential of 1,8-OH-AQ proved by the in vitro experiments encouraged the researchers to plan experiments on living organisms. Certainly, the number of undertaken experiments of this type has been growing within recent years. The major properties of 1,8-OH-AQ in BC in vivo models are set together in Table 2.

Emodin inhibited BC liver metastasis by regulating lipid synthesis in obese mice (fed with thigh fat diet for 8 weeks prior to the tumor injection) that were administered 400 mg/kg b.w., *p.o.* for 4 weeks. Interestingly, next to the anticancer effect, the compound exhibited regulatory properties towards the animal’s weight—it regulated the abnormal increase in the liver weight by its inhibitory properties towards cholesterol and fatty acids synthesis, fatty acids oxidation, and downregulation of triglycerides synthesis genes (*Fasn*, *Sed1*, *Gpat1*) [32].

Physcion was studied in the xenograft mice model of BC. Female BALB/c nude mice that were administered with 30 mg/kg of physcion, *i.p.* every day for 2 weeks, were found to have a suppressed tumor growth starting from the 8th day of treatment with no toxic effects observed on their body weight or organs’ structure. The anticancer activity could be related to the enhanced expression levels of Bax and cleaved caspase-3 but also to the suppressed expression levels of Bcl-xL, Bcl-2, Nrf2, HO-1, SOD-1, and SOD-2 proteins, suggesting an antioxidant mode of action. In further studies on immunosuppressive mice, the authors denote that physcion exhibits immunomodulatory action and is able to regulate the levels of interleukins, interferons, and tumor necrosis factors in a way to reduce the progression of BC [69].

The sensitization of known chemotherapeutics by rhein (10 mg/kg *i.p.)* was also confirmed by Shen et al., who studied the antiproliferative properties of atezolizumab (10 mg/kg *i.p.)* within BC treatment. The combination therapy on 4T1 BC xenografts in mice led to a strong reduction in the volume and weight of the tumor but also to an increase in the CD8+ T cells in the tumor and in the spleen. The serum levels of IL-6 and TNF-α were stimulated by rhein itself and in the rhein and atezolizumab group, leading to a conclusion that both rhein and atezolizumab—alone or in combination—are potent drugs for the treatment of 4T1 BC [94].

## 6. Sensitizing Properties of 1,8-Dihydroxyanthraquinones towards Known Drugs

As mentioned above, the derivatives of anthraquinones are often tested together with the other known chemotherapeutics. Anthraquinone derivatives of interest, namely, emodin, physcion, and rhein, were proven to interact with other drugs, leading to a more effective therapy. The sensitizing effects of 1,8-OH-AQ are particularly important since they can decrease the toxicity and diminish the side effects that often accompany standard therapeutical procedures. As shown in Table 2 and the previous sections, the safety profile of anthraquinone derivatives is promising. The doses as high as 400 mg/kg b.w. did not trigger any toxic effects on the soft organs or body weight of laboratory animals. Previously, scientists proved that the route of administration of an accompanying drug can differ from the main chemotherapeutic agent.

In recent years, combined therapy has become increasingly popular, which involves the simultaneous use of several substances, including compounds of natural origin. Combined therapy can lead to a reduction in toxicity to the patient’s body by replacing part of the dose of a conventional anticancer drug with a natural substance with known properties. Many studies emphasize that a combined approach may be more effective than using conventional chemotherapy agents alone.

The scientific literature lists examples that confirm stronger anticancer potential of a joint administration of the selected 1,8-dihydroanthraquinone derivatives with standard chemotherapeutics. The selected examples of this approach are listed in Table 3 below.

## 7. Discussion

Despite significant progress in the diagnosis and treatment of BC, this disease continues to pose a substantial global health challenge, impacting millions of women worldwide each year. Standard therapeutic options, which are commonly used in BC treatment, like radio- or chemotherapy, can result in a range of undesirable side effects that can significantly impact the quality of life for cancer patients. Additionally, the emergence of MDR in cancer cells can reduce the effectiveness of these therapies over time. Phytochemicals, which are natural compounds found in plants, hold promise in improving BC treatment outcomes. They have the potential to work synergistically with several chemotherapeutic agents, enhancing their effectiveness while minimizing some of the side effects associated with traditional therapeutic regimens. Anthracene derivatives are a group of tricyclic secondary metabolites that occur naturally and have been identified in various organisms, including plants, lichens, and fungi. They are promising compounds with proven anticancer properties. Mechanistic studies have established that the natural products derived from anthracene derivatives exert their anti-breast cancer activities through a wide array of molecular targets and mechanisms, including the modulation of angiogenesis, apoptotic pathways, autophagy, synthesis and repair genes expression, damage response, cell cycle regulators, EMT markers, epigenetic mechanisms, heat shock response, inflammation, metastasis-related markers, oxidative status, miRNA, protein synthesis, proliferation, or stem-like markers. The evolving insights into the tumor microenvironment and the underlying molecular pathways associated with BC create opportunities for the identification and development of new natural compounds that can be harnessed for their anticancer properties. These discoveries not only expand our arsenal of treatment options but also offer avenues for preventive strategies.

## 8. Conclusions

The review aimed to consolidate the information on 1,8-dihydroxyanthraquinones, including emodin, aloe-emodin, hypericin, chrysophanol, rhein, and physcion, as promising drug candidates for BC treatment. All these compounds, belonging to a subgroup of 1,8-dihydroanthraquinone derivatives, have been extensively studied for their anticancer properties. In the manuscript, the potential synergistic interactions between these natural compounds and commercially available chemotherapeutic agents in the in vitro and in vivo settings were also described. The evidence from the literature suggests that 1,8-dihydroxyanthraquinones indeed appear to be a promising group of natural compounds with potential effectiveness in the treatment of BC. This conclusion underscores their potential significance as candidates for further research and development in BC therapy.

## Figures and Tables

**Table 1:** The selected plant species containing anthracene derivatives.

Botanical Family	Gender Name	Selected Species
Rhamnaceae	*Rhamnus*, *Ventilago*	*Rhamnus purshiana*, *Rhamnus frangula*, *Rhamnus catartica*, *Ventilago maderaspatana*
Fabaceae	*Cassia*	*Cassia angustifolia*, *Cassia senna*, *Cassia obtusifolia*
Polygonaceae	*Rheum*, *Rumex*	*Rheum palmatum*, *Rheum officinale*, *Rheum rabarbarum* *Rumex conglumeratus*, *Rumex pulcher*, *Rumex alpinus*, *Rumex maritimus*, *Rumex obtusifolius*
Liliaceae	*Aloe*, *Bulbine*	*Aloe ferox*, *Aloe vera*, *Bulbine capitate*
Guttiferae	*Hypericum*	*Hypericum perforatum*
Rubiaceae	*Morinda*, *Rubia*, *Galium*, *Heterophyllea*	*Morinda citrifolia*, *Morinda officinalis* *Rubia tinctorum*, *Galium sinaicum*, *Galium verum*, *Heterophyllea pustulata*

**Table 2:** Anticancer properties of 1,8-dihydroxyanthraquinones in in vivo xenograft mice models (+—increase/stimulation, −—decrease/inhibition).

Compound	Cancer Type	Model	Dosing	Effect	References
Emodin	breast cancer MDA-MB-231 cells	nude BALB/c nu/nu mice fed a high-fat diet	400 mg/kg, *p.o.*, q.d. for 4 weeks	Regulation of body weight − cholesterol and fatty acids synthesis − fatty acids oxidation − *Fasn*, *Sed1*, *Gpat1* gene expression	[32]
Physcion	breast cancer	8 weeks, male BALB/c nude mice	30 mg/kg/day *i.p.* for 2 weeks	+ Bax and cleaved caspase-3, − Bcl-xL, Bcl-2, Nrf2, HO-1, SOD-1, and SOD-2 proteins expression in tumor tissue Immunomodulatory action	[69]
Rhein	breast cancer 4T1 cells	6–8 weeks, female BALB/c mice	10 mg/kg *i.p.* rhein every 3 days for a total of three times alone or with 10 mg/kg *i.p.* atezolizumab once every 2 days for a total of three times	With and without atezolizumab − tumor size (weight and volume) + CD8+ T cells production in the tumor and the spleen + serum levels of IL-6 and TNF-α	[94]

**Table 3:** Combinatorial effect of 1,8-dihydroanthraquinone derivatives with chemotherapeutics in breast cancer settings (+—increase/stimulation, −—decrease/inhibition).

Type of Cancer	1,8-Dihydroanthraquinone Derivatives	Chemotherapeutic	Effect	Ref.
Breast cancer	Rhein 10 mg/kg *i.p.* every 3 days, 3 times	Atezolizumab 10 mg/kg *i.p.* every 2 days, 3 times	− tumour size + CD8+ T cells production in the tumor and in the spleen + serum levels of IL-6 and TNF-α	[94]
Breast cancer	Emodin 110 µM	Doxorubicin 5 µM	Sensitisation to doxorubicin + apoptosis + DNA damage in cancer cells + γH2Ax expression − drug resistance to doxorubicin (PI3K-AKT pathway)	[95]
Breast cancer	Emodin 20 µM	5-Fluorouracil 40 µM	Sensitization to 5-fluorouracyl + apoptosis + ROS generation +cyclin-dependant kinase inhibitors expression − E2F1 expression − notch-regulated ankyrin repeat protein expression	[50]
Breast cancer	Physcion 5 mg/kg, *i.p.*	Paclitaxel 5 mg/kg, *i.p.*	Inhibition of 6PGD by physcion that results in the sensitization of breast cancer cells to paclitaxel	[96]

## Data Availability

No new data were created and analyzed in this manuscript. Data sharing is not applicable.
